# Alginate oligosaccharides enhance the antifungal activity of nystatin against candidal biofilms

**DOI:** 10.3389/fcimb.2023.1122340

**Published:** 2023-01-31

**Authors:** Lydia C. Powell, Jennifer Y. M. Adams, Sadik Quoraishi, Charlène Py, Anaϊs Oger, Salvatore A. Gazze, Lewis W. Francis, Christopher von Ruhland, David Owens, Philip D. Rye, Katja E. Hill, Manon F. Pritchard, David W. Thomas

**Affiliations:** ^1^ Advanced Therapies Group, Cardiff University School of Dentistry, Cardiff, United Kingdom; ^2^ Microbiology and Infectious Disease group, Swansea University Medical School, Swansea, United Kingdom; ^3^ Otolaryngology Department, New Cross Hospital, Wolverhampton, United Kingdom; ^4^ School of Engineering, University of Angers, Angers, France; ^5^ Centre for Nanohealth, Swansea University Medical School, Swansea, United Kingdom; ^6^ Central Biotechnology Services, Cardiff University School of Medicine, Cardiff, United Kingdom; ^7^ Head and Neck Directorate, University Hospital of Wales, Cardiff, United Kingdom; ^8^ AlgiPharma AS, Sandvika, Norway

**Keywords:** antifungal, alginate oligosaccharide, nystatin, *Candida* spp., biofilm

## Abstract

**Background:**

The increasing prevalence of invasive fungal infections in immuno-compromised patients is a considerable cause of morbidity and mortality. With the rapid emergence of antifungal resistance and an inadequate pipeline of new therapies, novel treatment strategies are now urgently required.

**Methods:**

The antifungal activity of the alginate oligosaccharide OligoG in conjunction with nystatin was tested against a range of *Candida* spp. (*C. albicans*, *C. glabrata*, *C. parapsilosis*, *C. auris*, *C. tropicalis* and *C. dubliniensis*), in both planktonic and biofilm assays, to determine its potential clinical utility to enhance the treatment of candidal infections. The effect of OligoG (0-6%) ± nystatin on *Candida* spp. was examined in minimum inhibitory concentration (MIC) and growth curve assays. Antifungal effects of OligoG and nystatin treatment on biofilm formation and disruption were characterized using confocal laser scanning microscopy (CLSM), scanning electron microscopy (SEM) and ATP cellular viability assays. Effects on the cell membrane were determined using permeability assays and transmission electron microscopy (TEM).

**Results:**

MIC and growth curve assays demonstrated the synergistic effects of OligoG (0-6%) with nystatin, resulting in an up to 32-fold reduction in MIC, and a significant reduction in the growth of *C. parapsilosis* and *C. auris* (minimum significant difference = 0.2 and 0.12 respectively). CLSM and SEM imaging demonstrated that the combination treatment of OligoG (4%) with nystatin (1 µg/ml) resulted in significant inhibition of candidal biofilm formation on glass and clinical grade silicone surfaces (*p* < 0.001), with increased cell death (*p* < 0.0001). The ATP biofilm disruption assay demonstrated a significant reduction in cell viability with OligoG (4%) alone and the combined OligoG/nystatin (MIC value) treatment (*p* < 0.04) for all *Candida* strains tested. TEM studies revealed the combined OligoG/nystatin treatment induced structural reorganization of the *Candida* cell membrane, with increased permeability when compared to the untreated control (*p* < 0.001).

**Conclusions:**

Antimicrobial synergy between OligoG and nystatin against Candida spp. highlights the potential utility of this combination therapy in the prevention and topical treatment of candidal biofilm infections, to overcome the inherent tolerance of biofilm structures to antifungal agents.

## Introduction

The increasing prevalence of invasive fungal infections in immuno-compromised patients such as the elderly, those undergoing organ transplantation/cancer treatment (Miceli et al., 2011; Vallabhaneni et al., 2016), suffering with COVID-19 (Hoenigl et al., 2022) or having implanted medical devices (Kojic and Darouiche, 2004; Buijssen et al., 2012) represents a considerable cause of morbidity and mortality (Low and Rotstein, 2011). This global health threat is further compounded by the under-recognized emergence of the antifungal resistance crisis, recently highlighted in the World Health Organisation fungal priority pathogens list (WHO, 2022). Currently, only four classes of systemic antifungal medicines (azoles, polyenes, echinocandins and pyrimidines) are routinely used in clinical practice (Brown et al., 2012; Denning, 2022). While azole antifungals are normally the preferred treatment for *Candida* infections, many *Candida* spp. already display intrinsic or have acquired resistance to azole treatment (Whaley et al., 2017; Fisher et al., 2022). The loss of echinocandin activity has also been demonstrated following prolonged caspofungin treatment for *C. albicans* oesophagitis (Fisher et al., 2022). The recent global spread of the multidrug-resistant pathogen *Candida auris*, which causes severe invasive infections associated with high mortality and transmissibility rates, combined with long-term survival on surfaces, highlights a growing problem (Du et al., 2020; Ademe and Girma, 2020).

Candidal cells can grow and exist within a three-dimensional complex mesh of self-derived extracellular polymers known as biofilms, which further enhances their pathogenicity. These biofilms demonstrate increased tolerance to antifungal agents and host immune defenses through expression of virulence factors, reduced penetration, sequestration of antifungal agents and development of persister cells (Taff et al., 2013; Cavalheiro and Teixeira, 2018). Importantly, *Candida* species exhibit the propensity to form biofilms on implanted medical devices such as urinary and vascular catheters, mechanical heart valves, joint prostheses and tracheoesophageal prostheses. These biofilms can lead to bloodstream infections, with nearly 80% of patients suffering from invasive candidiasis found to possess an implanted medical device (Andes et al., 2012; Nobile and Johnson, 2015; Eix and Nett, 2020).

The polyene agent, nystatin, has been used topically to treat infections on implanted devices (Ameye et al., 2005; Bodin et al., 2015; Brook and Goodman, 2020) and for cutaneous and vulvovaginal candidiasis (Dressen et al., 2012; Patel et al., 2017), as well as being used as an oral formulation to treat oral and intestinal candidosis (Lyu et al., 2016). A study of candidal isolates from tracheoesophageal prostheses demonstrated a broad range of minimum inhibitory concentrations (MICs) for miconazole (0.062 to 64 g/mL) whilst MIC values for nystatin were more narrowly distributed (2 to 8 g/mL), reflecting the greater sensitivity of *Candida* colonizing voice prostheses to nystatin (Bauters et al., 2002). However, significant increases in nystatin resistance in *Candida* spp. have been demonstrated (by up to 16-fold) in biofilms when compared to planktonic assay systems (Chandra et al., 2001). Therefore, the treatment of candidal biofilms represents a complex clinical and therapeutic challenge, especially in the emerging antifungal resistance crisis, where new treatment strategies are now urgently required to overcome such infections.

The low molecular weight alginate oligosaccharide, OligoG, composed of >85% α-ʟ-guluronic acid oligomers, has shown potential as an antifungal agent. OligoG has demonstrated the ability to both inhibit growth of *Candida* spp. and potentiate the effects of the antifungal agents (nystatin and fluconazole) against *C. albicans*, *C. parapsilosis*, *C. krusei*, and *C. tropicalis*, with an up to 16-fold observed reduction in MIC when used as a dual therapy (Tøndervik et al., 2014). OligoG has also been found to reduce *in vitro* virulence factor expression and hyphal invasion by *C. albicans* (Pritchard et al., 2017).

Previous studies only evaluated the antifungal activity of OligoG on planktonic cells and *C. tropicalis* biofilms. In this study we assessed the *in vitro* ability of OligoG alone, and in combination with nystatin, to inhibit planktonic growth, perturb biofilm formation on glass and clinical grade silicone surfaces and disrupt established biofilms of a wide range of candidal clinical isolates currently on the WHO fungal priority pathogens list.

## Materials and methods

### Microbial strains and growth conditions

A total of thirteen *Candida* strains were selected for this study; eight of which were *Candida albicans* and five non-*Candida albicans Candida* (NCAC) as shown in **Table 1**. Identification of the *Candida* species used in this study was confirmed by MALDI-TOF mass spectrometry. For all assays, *Candida* strains were grown on Sabouraud Dextrose (SAB; Lab M) agar at 37°C and in Roswell Park Memorial Institute medium (RPMI 1640; Sigma-Aldrich; Clinical and Laboratory Standards Institute [CLSI], 2002) for overnight liquid culture (O/N). Unless stated, strains were grown at 37°C (120 rpm shaking) and adjusted with RPMI 1640 to a standardized cell suspension of 5x10^6^ cells/ml (OD_600_ 0.37). Nystatin (Sigma-Aldrich) was prepared in dimethyl sulfoxide (DMSO; 5 mg/ml). The low molecular weight alginate OligoG was provided by AlgiPharma AS and prepared, purified, and characterized as previously described (Khan et al., 2012). Clinical grade silicone sheets of the same material used in the manufacture of Provox tracheo-esophageal prostheses were obtained from Atos Medical (Nottingham, UK).

**Table 1:** *Candida* strains, sources, and MIC (μg/ml) of nystatin alone and with increasing concentrations of OligoG (0.5, 1, 2, 4 and 6%; n=3).

Isolate	Isolation Source	Reference	Antifungal MIC (μg/ml) at indicated OligoG Concentration (%)					
			0	0.5	1	2	4	6
*C. albicans* ATCC 90028	Blood	ATCC	2	2	2	1	1	0.5
*C. albicans* GBJ 13/4A	Failed TE prosthesis	Elving et al., 2000	2	2	2	2	1	1
*C. albicans* SC5314	Human clinical specimen	ATCC	1	1	1	1	0.5	0.5
*C. albicans* CCUG 39343	Human faeces	CCUG	2	2	2	1	1	1
*C. albicans* 480/00	SCC, oral mucosa	Bartie et al., 2004	2	4	4	2	1	1
*C. albicans* PB1/93	Normal oral mucosa	Bartie et al., 2004	2	2	4	2	1	1
*C. albicans* Lr1/93	Normal oral mucosa	Bartie et al., 2004	1	2	2	1	0.5	0.5
*C. albicans* Ptr/94	CHC, buccal mucosa	Bartie et al., 2004	2	2	2	2	2	2
*C. auris* NCPF 8971	Wound swab	NCPF	2	4	4	4	2	2
*C. dubliniensis* 40/01^a^	PMC, palate	Bartie et al., 2004	2	2	2	0.5	0.25	0.063
*C. glabrata* ATCC 2001	Faeces	ATCC	4	8	4	4	2	2
*C. parapsilosis* W23	Human oral specimen	This Study	4	2	2	2	1	1
*C. tropicalis* 519468	Urinary tract	Silva et al., 2009	2	2	1	1	1	1

TE, tracheo-esophageal; CHC; chronic hyperplastic candidosis, SCC; squamous cell carcinoma, PMC; pseudomembranous candidosis, ATCC; American Type Culture Collection, NCPF; The National Collection of Pathogenic Fungi; CCUG; Culture Collection University of Gothenburg.

a formally known as C. albicans 40/01.

### Minimum inhibitory concentration assays

MIC assays for the *Candida* strains were performed according to the CLSI (2002) guidelines. In flat-bottomed 96-well microtiter plates, two-fold serial dilutions of nystatin (0.0156 – 16 µg/ml) were prepared in RPMI 1640 ± OligoG (0.5, 1, 2, 4, and 6%). Five fungal colonies from the plate were adjusted to a standardized cell suspension of 1x10^6^ cells/ml in phosphate buffered saline (PBS). The adjusted culture was further diluted 1:50 in PBS and then diluted again 1:1 in RPMI 1640 medium to produce the inoculum. A 5 µl inoculum of the adjusted cultures was then added to each well and plates were incubated statically for 48 h at 37°C (n=3). The MIC values were determined as the modal value of the lowest concentration at which there was no visible growth.

### Growth curve assay

O/N adjusted *Candida* cultures (1x10^6^ cells/ml) were diluted in RPMI 1640 (1:10) in 96-well plates with ½ MIC nystatin (1-2 µg/ml) treatment ± OligoG (2, 4, 6%) and grown for 48 h at 37°C (n=3). The absorbance (OD_600_) was measured hourly using a FLUOstar Omega plate reader (BMG Labtech), with results presented as mean values. The minimum significant difference (MSD) was calculated using the Tukey-Kramer method using Minitab 17.2.1 (Minitab Inc, State college, PA, USA).

### Confocal laser scanning microscopy biofilm formation assay

Adjusted O/N *Candida* cultures (5x10^6^ cells/ml) were incubated in a black, glass-bottomed 96-well plate (Greiner) for 45 min with prewarmed RPMI 1640 medium in a 1:4 ratio (37°C, 20 rpm rocking). After incubation, the wells were washed with pre-warmed RPMI 1640 (3x) before adding fresh RPMI 1640 ± 4% OligoG ± 1 µg/ml nystatin to each well, before a further 24 h incubation (37°C, 20 rpm; n=3). After biofilm formation, all the supernatant was removed and biofilms stained with 0.4% (v/v) LIVE/DEAD^®^ stain in PBS (BacLight™ Bacterial Viability kit; Invitrogen), before imaging using a Leica TCS SP5 confocal laser scanning microscope (CLSM). The green SYTO 9 stain was used to visualize LIVE cells (excitation/emission 480/500 nm), while red propidium iodide (PI; excitation/emission 490/635 nm) was used to visualize the DEAD/dying cells.

### Scanning electron microscopy biofilm formation assay

O/N adjusted *C. parapsilosis* W23 and *C. albicans* GBJ 13/4A cultures (1x10^6^ cells/ml) were first added to a 12-well plate containing silicone samples, followed by pre-warmed RPMI 1640 (W23) or ½ SAB medium (GBJ 13/4) in the ratio 1:5 and incubated rocking at 20 rpm at 37°C. After 45 min incubation, the resultant biofilms were washed in the appropriate pre-warmed medium (x3), before 1 ml of further medium was added ± nystatin (1 µg/ml) ± OligoG (4%), with a further 24 h incubation on a rocker at 20 rpm at 37°C (n=3). The supernatant was removed, and biofilms were fixed with 2.5% (v/v) glutaraldehyde for 1.5 h. Following washing (x4) with dH_2_O, the fixed biofilms were covered with 1 ml dH_2_O, frozen and freeze-dried. The samples were then gold–coated and imaging performed using a Tescan Vega3 scanning electron microscope (SEM) at 6 kV.

### Confocal laser scanning microscopy biofilm disruption assay

O/N adjusted *Candida* cultures (5x10^6^ cells/ml) were incubated for 45 min within a glass-bottomed 96-well plate (Grenier). After incubation, the wells washed with pre-warmed RPMI 1640 (x3) and 200 µl of RPMI 1640 medium added into each well, before a further 24 h incubation (20 rpm rocking, 37°C). After 24 h, half the supernatant was removed and replaced with 100 µl of fresh medium containing ± OligoG (8%) and nystatin (4 µg/ml) (final concentration of 4% OligoG and 2 µg/ml nystatin) with a further 24 h incubation (20 rpm rocking, 37°C; n=3). After 24 h, the supernatant was removed, and biofilms stained with LIVE/DEAD^®^ BacLight™ stain (Invitrogen) for 10 min (as performed in the Biofilm Formation assay) prior to CLSM imaging using a Leica TCS SP5 CLSM.

### COMSTAT analysis

CLSM z-stack images were analyzed using COMSTAT image analysis software for quantification of specific parameters of the three-dimensional biofilm structure including bio-volume and DEAD/LIVE cell ratio (Heydorn et al., 2000).

### ATP biofilm disruption assay

O/N adjusted *Candida* cultures (5x10^6^ cells/ml) were added to a black walled flat-bottomed 96-well plate (Grenier), followed by pre-warmed RPMI 1640, in a 1:10 ratio and incubated (20 rpm, 37°C). After 45 min incubation, the resultant biofilms were washed with pre-warmed RPMI 1640 (x3), before adding a further 200 µl of fresh medium and incubating for a further 24 h (20 rpm, 37°C). Biofilm disruption was then assessed by removing half of the supernatant from each well and replacing it with fresh RPMI 1640 ± 4% OligoG (v/v) ± MIC nystatin (v/v; 1-4 µg/ml) followed by a further 24 h incubation (37°C, 20 rpm; n=3). To assess biofilm disruption after treatment, the BacTiter-Glo™Microbial Cell Viability Assay (Promega) was used to determine luminescence as measure of viable fungal cell number, using a FLUOstar Omega plate reader.

### Ergosterol assay

*Candida* MIC assays were repeated in the presence and absence of ergosterol in a 96-well microtiter plate (100 µl/well). Ergosterol was dissolved in DMSO containing 1% Tween 80 (Sigma-Aldrich) prior to being diluted to a final concentration of 400 µg/ml in RPMI 1640. Plates were read visually after 2 days incubation at 37°C (n=3). The antifungal Amphotericin B was used as a positive control.

### Permeabilization assay

O/N adjusted *C. parapsilosis* W23 cultures (1x10^7^ cells/ml) was first added to a black glass-bottomed 96-well plate (Grenier) followed by pre-warmed RPMI 1640 in the ratio 1:10 and incubated shaking for 24 h at 20 rpm (37°C). After incubation, each well was washed with dH_2_O, after which 200 µl of RPMI 1640 ± nystatin (1 µg/ml) ± OligoG (4%) was added to each well and incubated for 3 h at 37°C (n=3). Samples were then washed and exposed to PI (1 mg/ml) for 15 min at 37°C, washed and mounted using Vectashield^®^ prior to examination by Leica TCS SP5 CLSM microscopy. Fungal membrane permeability was assessed by measuring the fluorescence intensity of PI from CLSM z-stack imaging using the IMARIS software.

### Transmission electron microscopy imaging of the candidal cell wall

Adjusted O/N cultures (5x10^6^ cells/ml) of *C. parapsilosis* W23 were inoculated onto thermanox cover slips and grown for 45 min (37°C, 20 rpm rocking) in 6-well microtiter plates. After incubation, the biofilms were washed with prewarmed RPMI 1640 (3x), before adding 2 ml of medium ± nystatin (1 µg/ml) ± OligoG (4%). Plates were then incubated on a rocker for 24 h (37°C, 20 rpm; n=1). After incubation, the supernatant was removed, and cells were fixed with 1% glutaraldehyde for 1 h before replacing the fixative with PBS. Fixed cells were centrifuged at 100 *g* for 10 min before the pellet was mixed with 4% molten agarose in equal volumes and was cooled to room temperature. The blocks were cut into 1 mm cubes and washed in double distilled water (ddH_2_O) for 10 min before being processed into resin in a rotary mixer at room temperature by the following steps: Post-fixed for 1 h in 2% (w/v) osmium tetroxide in ddH_2_O, 10 min wash in ddH_2_O (x3), block stained for 1 h in 2% uranium acetate in ddH_2_O, 10 min wash in ddH_2_O (x3), 15 min in 50% (v/v) propan-2-ol (IPA) in ddH_2_O, 15 min in 70% (v/v) IPA in ddH_2_O, 15 min in 90% (v/v) IPA in ddH_2_O, 15 min in IPA (2x), 30 min in 50% (v/v) hard grade TAAB Embedding Resin (TER) in IPA, 1 h in TER (4x). Blocks were transferred into truncated polypropylene BEEM capsules and the capsules filled with fresh TER resin. Capsules were placed in a 60°C oven and curing allowed to proceed for 24 h. Semi-thin (0.35 mm thick) sections were cut with glass knives on an Ultracut E ultramicrotome (Leica Microsystems), placed on droplets of water on a glass slide and dried on a hot plate. Sections were stained with 1% toluidine blue, washed in tap water, dried, and mounted with Gurr’s Neutral Mountant. Thin (100 nm thick) sections were cut with a diamond knife on an Ultracut E ultramicrotome, collected onto 300 mesh copper grids and allowed to air dry. Sections were stained with Reynold’s lead citrate for 15 min, washed in ddH_2_O for 1 min (3 x) and allowed to air dry. Samples were examined in a Hitachi HT7800 transmission electron microscope (TEM) (Hitachi High Tech Ltd; UK) at 100 kV and images captured with Radius software (EMSIS GmbH; Germany).

## Results

### OligoG enhanced the antifungal effect of nystatin in MIC assays

The MIC assays revealed that the presence of OligoG enhanced the antifungal effect of nystatin against a range of *Candida* species (**Table 1**). The greatest dose-dependent effect was seen for *C. dubliniensis* 40/01, where there was a 32-fold decrease in MIC value when nystatin was used in combination with 6% OligoG. *C. albicans* ATCC 90028 and *C. parapsilosis* W23 showed a 4-fold decrease in MIC value. The remaining ten strains demonstrated no effective change in MIC value with either a minimal 2-fold reduction or no change at all between 0% and 6% OligoG.

### The combination treatment of OligoG and nystatin reduced candidal growth

**Figure 1:**
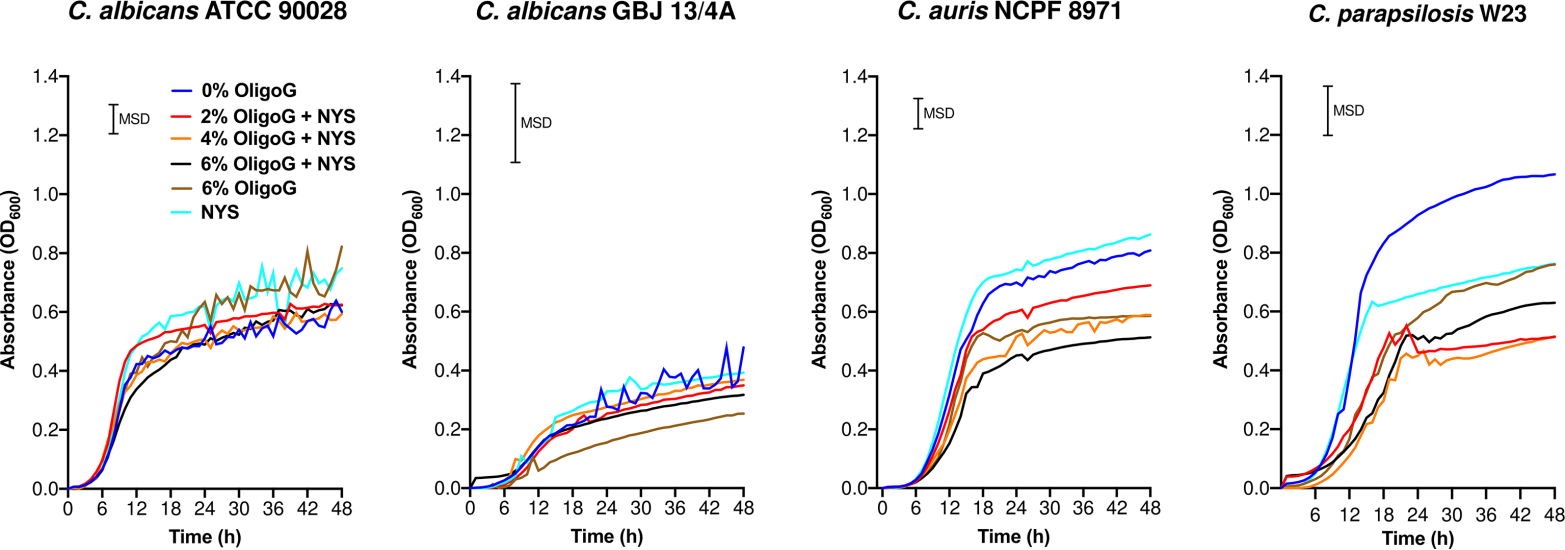
Growth curves for *Candida* spp. treated with OligoG (2, 4 and 6%) in combination with nystatin (NYS; at ½ MIC; 1-2 µg/ml). Minimum significant difference (MSD) for absorbance was calculated in MiniTab using the Tukey-Kramer method (n=3): *C. albicans* ATCC 90028 (MSD=0.12), *C. albicans* GBJ 13/4A (MSD=0.32), *C. auris* NCPF 8971 (MSD=0.12) and *C. parapsilosis* W23 (MSD= 0.20).

Four *Candida* strains were selected for further analysis, based on their listing in the critical (*C. albicans* and *C. auris*) and high (*C. parapsilosis*) priority groups in the WHO fungal priority pathogens list and also their propensity to form biofilms on implant surfaces (Bauters et al., 2002; Yamin et al., 2021; WHO, 2022). The growth curves of the selected *Candida* strains revealed that OligoG alone (tested at 0 and 6%) showed a significant inhibition of growth for *C. parapsilosis* W23 and *C. auris* NCPF 8971 (0 – 48 h; MSD = 0.20 and 0.12 respectively) when compared to the untreated control, although this inhibition was not evident for *C. albicans* ATCC 90028 or *C. albicans* GBJ 13/4A (**Figure 1**; MSD = 0.12 and 0.32 respectively). The addition of OligoG to nystatin treatment markedly impaired *C. parapsilosis* W23 growth (at 2 and 4% OligoG) and *C. auris* NCPF 8971 growth (at 2, 4 and 6% OligoG) when compared to the nystatin-only control, overall growth at 48 h (**Figure 1**). However, the combination treatment did not significantly alter 48 h overall growth of the strains *C. albicans* GBJ 13/4A and *C. albicans* ATCC 90028.

### The combination treatment of OligoG and nystatin inhibited biofilm formation of *Candida* spp.

**Figure 2:**
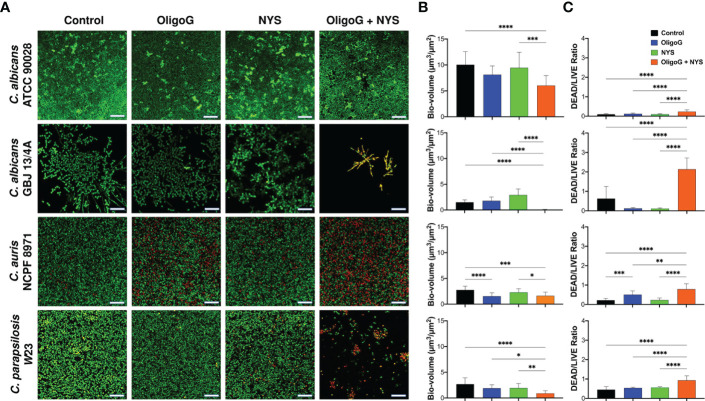
CLSM imaging of *C. albicans* ATCC 90028, *C. albicans* GBJ 13/4A, *C. auris* NCPF 8971 and *C. parapsilosis* W23 biofilm formation (24 h) treated with 4% OligoG in combination with 1 μg/ml nystatin (NYS). **(A)** Z-stack imaging; scale bar = 40 μm. Corresponding COMSTAT analysis of **(B)** biofilm biovolume (µm^3^/µm^2^) and **(C)** DEAD/LIVE bacterial ratio (n=3). Group wise comparisons were analysed using one-way ANOVA followed by Tukey’s *post hoc* tests *p < 0.05; **p < 0.01; ***p < 0.001; ****p < 0.0001.

The CLSM biofilm formation assay performed with *C. albicans* ATCC 90028, *C. albicans* GBJ 13/4A, *C. auris* 8971 and *C. parapsilosis* W23 revealed that the combination treatment (4% OligoG and 1 µg/ml nystatin) markedly inhibited biofilm formation (**Figure 2A**). COMSTAT analysis of the CLSM images revealed significant reductions in biofilm biovolume (**Figure 2B**), significant increases in the DEAD/LIVE bacterial ratio (**Figure 2C**) and significant increases in biofilm roughness (**Supplementary Figure 1A**) when compared to the untreated control and nystatin-only treatment for all *Candida* strains tested (p < 0.05). Interestingly, OligoG-only treatment was highly effective against *C. auris* NCPF 8971 biofilm formation, with a significant reduction in biofilm biovolume and a significant increase in DEAD/LIVE bacterial ratio when compared to the untreated control (p < 0.001).

### The combination treatment of OligoG and nystatin inhibited biofilm formation of *Candida* spp. grown on silicone surfaces

**Figure 3:**
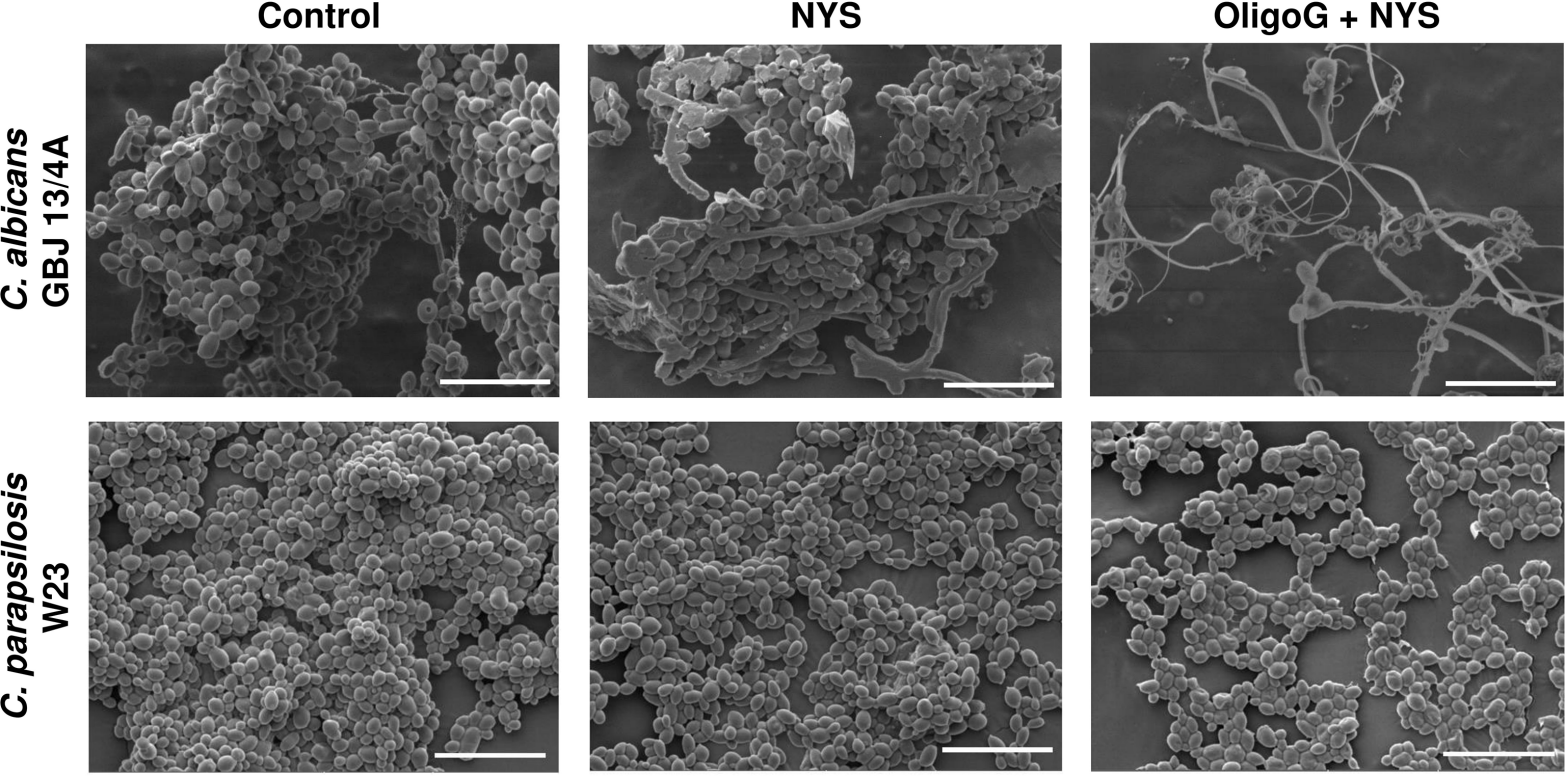
SEM imaging of *C. albicans* GBJ 13/4A and *C. parapsilosis* W23 biofilm formation on silicone surfaces treated with 4% OligoG in combination with 1 µg/ml nystatin (NYS) after 24 h growth (n=3); scale bar = 20 μm.

SEM imaging of *C. albicans* GBJ 13/4A and *C. parapsilosis* W23 biofilms grown on silicone surfaces (24 h) revealed that the combination of OligoG/nystatin treatment significantly modified the biofilm structure (**Figure 3**). For *C. parapsilosis* W23, there appeared to be a reduction in the multi-layered 3-dimensional biofilm structure to a markedly reduced, more homogeneous, “flattened” architecture on the silicone surface after the combination treatment. In contrast, for *C. albicans* GBJ 13/4A, a less adherent biofilm with markedly fewer cells was evident following the combination treatment.

### OligoG in combination with nystatin treatment disrupted the structure and reduced cellular viability in established *Candida* biofilms

**Figure 4:**
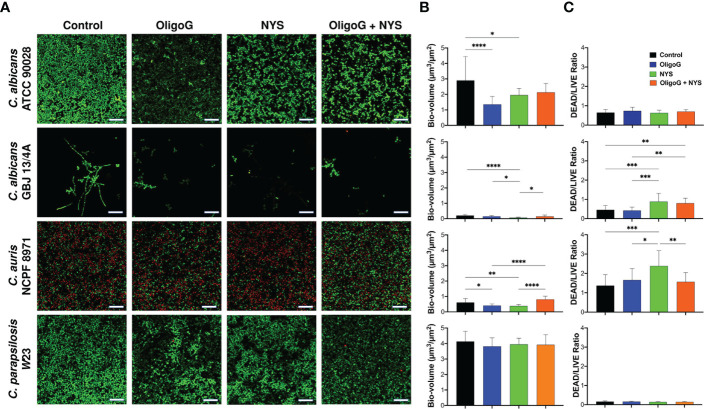
CLSM imaging of the disruption of 24 h grown *C. albicans* ATCC 90028, *C. albicans* GBJ 13/4A, *C. auris* NCPF 8971 and *C. parapsilosis* W23 established biofilms treated with 4% OligoG ± 2 μg/ml nystatin (NYS) for a further 24 h. **(A)** Z-stack imaging; scale bar = 40 μm. Corresponding COMSTAT analysis of **(B)** biofilm biomass (µm^3^/µm^2^) and **(C)** DEAD to LIVE bacterial ratio; (n=3). Group wise comparisons were analysed using one-way ANOVA followed by Tukey’s *post hoc* tests *p < 0.05; **p < 0.01; ***p < 0.001; ****p < 0.0001.

The CLSM biofilm disruption assay performed with *C. albicans* ATCC 90028, *C. albicans* GBJ 13/4A, *C. auris* NCPF 8971 and *C. parapsilosis* W23 revealed that the combination treatment (4% OligoG + 2 µg/ml nystatin) visibly altered the biofilm architecture, inducing a more porous structure (**Figure 4A**). However, COMSTAT analysis did not reveal any significant gross structural alterations in biofilm biovolume when compared to the untreated control (**Figure 4B**; p > 0.05). Only *C. albicans* GBJ 13/4A revealed a significant increase in DEAD/LIVE cell ratio for the combination treatment when compared to the untreated control (**Figure 4C**; p < 0.01) however, this cell ratio was not significant when compared to nystatin-only treatment (p > 0.05). Only *C. albicans* GBJ 13/4A and *C. albicans* ATCC 90028 demonstrated a significant increase in biofilm roughness for the combination treatment when compared to the untreated control (**Supplementary Figure 1B**; p < 0.001) however, this roughness was not significant when compared to nystatin-only treatment (p > 0.05). Interestingly, *C. albicans* ATCC 90028 and *C. auris* NCPF 8971 exhibited a significant reduction in biofilm biovolume with OligoG-only treatment when compared to the untreated control (p < 0.0001 and p < 0.05 respectively).

**Figure 5:**
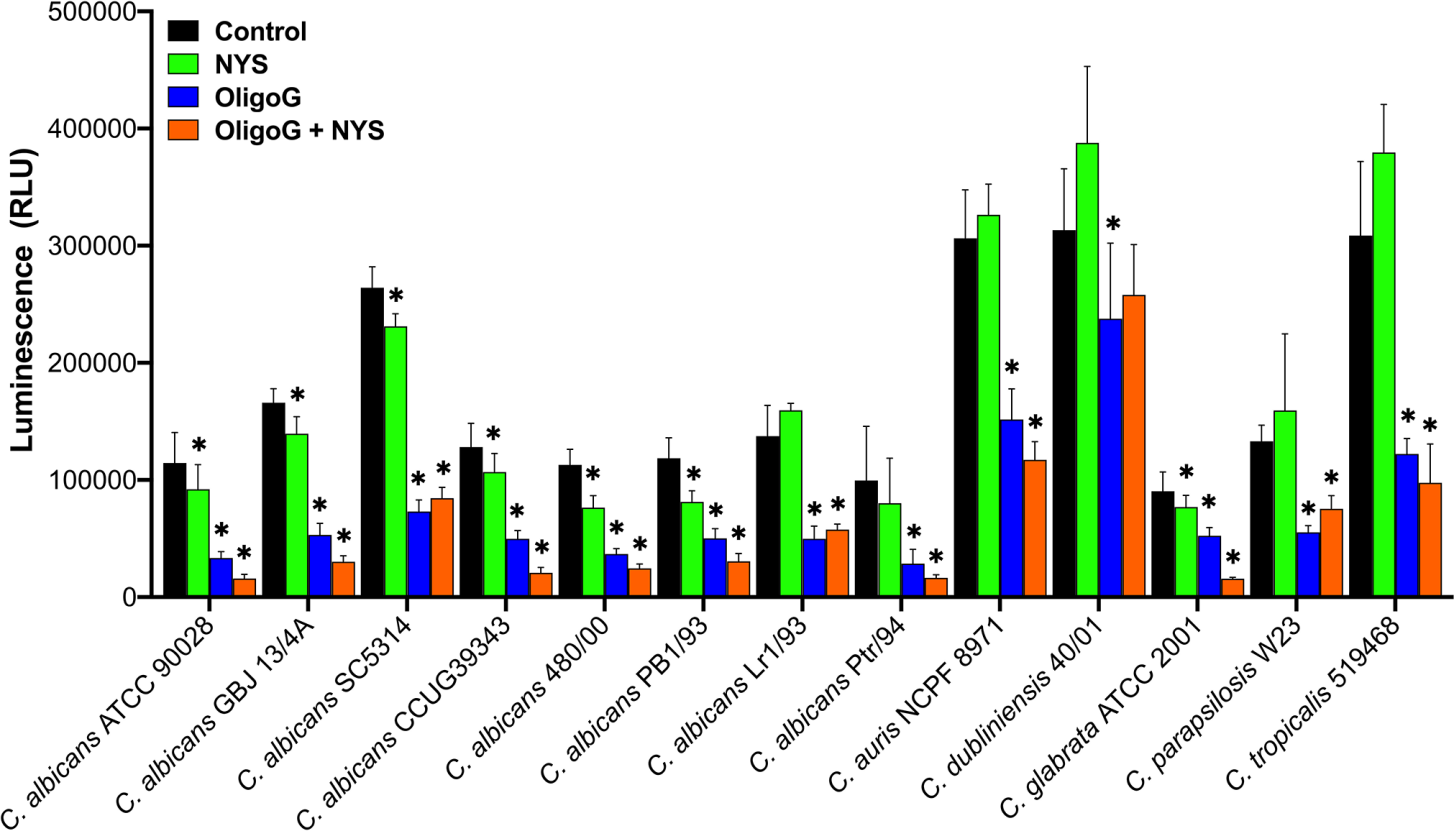
ATP cellular viability assay for biofilms of thirteen *Candida* spp. grown for 24 h prior to treatment with ± 4% OligoG ± nystatin (NYS; at MIC, 1-4 μg/ml) for a further 24 h (n=3). Group wise comparisons were analysed using one-way ANOVA followed by Tukey’s *post hoc* tests; * p < 0.05 compared to an untreated control. (All p values are shown in **Supplementary Table 1**).

To obtain a more global measure of biofilm disruption with a wide range of *Candida* strains, an ATP assay was used to assess the ability of the combination treatment (24 h treatment with 4% OligoG and MIC value of nystatin) to reduce the cellular viability of 24 h pre-established biofilms. The ATP assay demonstrated significant reductions in cellular viability when OligoG was used in combination with nystatin compared to the untreated control and nystatin-only treatments for all strains (p < 0.006; **Figure 5**, **Supplementary Table 1**), except for *C. dubliniensis* 40/01. Interestingly, the ATP assay also demonstrated significant reductions in cellular viability when the OligoG-only treatment was compared to the untreated control and nystatin-only treatments for all thirteen *Candida* strains (p < 0.04; **Figure 5**, **Supplementary Table 1**).

### OligoG enhanced the antifungal effect of nystatin in the presence of additional ergosterol within the MIC assays

To assess whether enhancement of the antifungal effect of nystatin by OligoG was due to potentiation of nystatin’ through enhanced ergosterol binding, MIC assays were performed with added ergosterol (**Table 2**). The binding of nystatin to exogenous ergosterol present in the medium should be reflected by an observed increase in MIC value in comparison to the no ergosterol control (de Castro et al., 2015). As expected, the addition of ergosterol to the nystatin-only treatment resulted in a 4-fold increase in the MIC value for *C. albicans* GBJ 13/4A and *C. auris* NCPF 8971 and a 2-fold increase in MIC value for *C. albicans* ATCC 90028. Furthermore, if OligoG enhanced nystatin’s affinity for ergosterol binding, the presence of exogenous ergosterol in these assays should minimize changes in the MIC value for the combination treatment in comparison to nystatin-only (plus ergosterol) treatment. However, this was not the case, as the MIC of the combination treatment (6% OligoG plus nystatin) in the presence of additional ergosterol demonstrated a decrease (up to 8-fold) compared to the nystatin-only (plus ergosterol) treatment.

**Table 2:** MIC values (µg/ml) of the antifungal nystatin at indicated OligoG concentrations (0.5, 1, 2, 4, 6%) in the presence and absence of ergosterol (400 µg/ml) against *Candida* strains measured after 2 days incubation (n=3).

Isolate	Antifungal MIC (μg/ml) at indicated OligoG Concentration (%)													
	0		0.5		1		2		4		6		AmB	
	-	+	-	+	-	+	-	+	-	+	-	+	-	+
*C. albicans* ATCC 90028	4	8	4	8	4	8	4	8	2	2	2	2	<0.0625	16
*C. albicans* GBJ 13/4A	2	8	2	8	2	8	2	8	0.5	4	0.5	1	<0.0625	16
*C. auris* NCPF 8971	4	16	4	16	4	16	4	16	4	8	2	4	<0.0625	16
*C. parapsilosis* W23	4	4	4	4	4	4	4	4	4	4	2	2	<0.0625	2

AmB, amphotericin B. #-, without ergosterol; +, with ergosterol.

### OligoG in combination with nystatin did not increase permeabilization of *C. parapsilosis* W23 in comparison to nystatin alone

**Figure 6:**
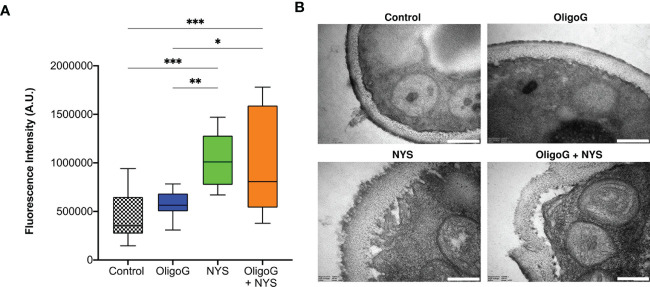
**(A)** Fluorescence intensity data derived from PI staining used in the fungal membrane permeability assay of *C. parapsilosis* W23 treated ± 4% OligoG ± 1 µg/ml nystatin (NYS; n=3). Group wise comparisons were analysed using one-way ANOVA followed by Tukey’s *post hoc* tests *p < 0.05; **p < 0.01; ***p < 0.001. **(B)** TEM imaging of *C. parapsilosis* W23 treated ± 4% OligoG ± 1 µg/ml nystatin, demonstrating disruption of the cell wall with nystatin treatment; scale bar 200 nm (n=1).

The fungal membrane permeability assay revealed unsurprisingly that nystatin treatment significantly increased cell permeabilization when compared to the untreated control (p < 0.001; **Figure 6A**). Whilst the combination treatment of OligoG and nystatin still demonstrated significantly increased cell permeabilization when compared to the control (p < 0.001), there was no significant difference (p > 0.05) in cell permeabilization when comparing the combination treatment to the nystatin-only treatment. TEM imaging showed that both the nystatin-only treatment and the combination treatment induced structural reorganization of the *Candida* cell membrane (**Figure 6B**), which was not evident in the untreated control or OligoG-only treatments.

## Discussion

With the clinical emergence of antifungal resistance particularly in immuno-compromised patients, and in the environment due to fungicide use in agriculture, the development of alternative treatment strategies to combat antifungal resistance in fungal infections is of vital importance (Fisher et al., 2022; WHO, 2022). This study demonstrated the ability of OligoG to act synergistically with nystatin to overcome the tolerance of candidal biofilms to antifungal treatments (Taff et al., 2013). Previous studies have revealed significantly decreased susceptibility to nystatin in *C. albicans* and *C. parapsilosis* biofilms when compared to planktonic forms (Kuhn et al., 2002). These increased levels of antifungal resistance in *Candida* biofilms have (at least in part) been shown to be due to up-regulation of efflux pumps (Ramage et al., 2002), gene mutations in drug-target enzymes e.g. *ERG11* (azoles) and *FKS1* (echinocandins; Taff et al., 2013) as well as sequestration of antifungals within the β-glucan biofilm matrix (Vediyappan et al., 2010). Furthermore, Mukherjee et al. (2003) demonstrated the importance of ‘time-specific efflux pump functionality’ showing that the highest levels of transcription and, therefore, efflux pump expression occurred early in biofilm development, and that this was a coordinated response of several efflux pumps causing increased levels of resistance. The same study also revealed that ergosterol levels in the cell membranes of biofilm-growing cells decreased with biofilm age compared to planktonic cells, which could lead to reduced efficacy of ergosterol-targeting drugs, such as nystatin, on more mature biofilms.

This *in vitro* study clearly demonstrated the ability of the alginate oligosaccharide, OligoG, to synergistically enhance the action of a conventional antifungal therapy resulting in reduced cellular growth, inhibition of biofilm formation and disruption of established biofilms of *Candida* strains. Interestingly, there was an up to 32-fold reduction in the MIC values of *Candida* strains when treated with nystatin in combination with OligoG (6%) and the growth curve assay revealed that the combination treatment of OligoG (2 - 6%) and nystatin (at ½ MIC) resulted in a significant inhibition of growth for *C. auris* NCPF 8971 and *C. parapsilosis* W23, although this was not evident for *C. albicans* ATCC 90028 or *C. albicans* GBJ 13/4A when compared to the controls.

Inhibition of *Candida* biofilm formation for all strains grown on glass or clinical grade silicone substrate was evident for the OligoG/nystatin combination treatment. The ATP biofilm disruption assay also demonstrated a marked reduction in *Candida* cellular viability with both OligoG alone and the combined OligoG/nystatin therapy. This study demonstrates the ability of the combination treatment to overcome the inherent tolerance within candidal biofilms to antifungal agents. Consequently, this could allow reduced dosages of antifungal agents to be used for biofilm-related infections, such as those associated with silicone-based implanted medical devices, thereby also effectively reducing the innate toxicity of such treatments.

Mechanistically, nystatin binds to membrane sterols such as ergosterol (a major component of the fungal cell wall) altering the permeability of the cell membrane, leading to leakage of intracellular contents and ultimately resulting in cell death (Hąc-Wydro and Dynarowicz-Łątka, 2006). This study demonstrated that the effects seen here arising from the combined treatment of OligoG/nystatin in *C. parapsilosis* W23 (compared to nystatin alone) were not a result of increased cell permeabilization and cell membrane disruption. Furthermore, the ergosterol assay revealed that even in the presence of additional ergosterol, the MIC of the combination treatment of OligoG/nystatin decreased in comparison to the nystatin-only (plus ergosterol) treatment. This data suggests that a secondary mechanism of action, other than ergosterol binding, contributes to the marked antifungal effects noted in this study.

Naturally occurring alginates are composed of high molecular weight β-D-mannuronic acid (M) and α-L-guluronic acid (G) linear polymers; the conformational arrangement of the G-block co-polymer having a strong Ca^2+^-binding affinity (Borgogna et al., 2013), especially when compared to M- and MG-blocks (Smidsrød, 1974; Lattner et al., 2003). OligoG is a low molecular weight oligomer, with a high G-residue composition greather than 85%, and as such, displays strong binding affinity to calcium. The ability of OligoG to chelate calcium has been hypothesized as a mechanism by which it is able to modify the assembly of cystic fibrosis intestinal mucus and disrupt the extracellular polymeric network of mucoid *P. aeruginosa* biofilms (Vitko et al., 2016; Ermund et al., 2017; Powell et al., 2018).

Previous studies have shown that calcium-chelating agents, such as ethylenediaminetetraacetic acid (EDTA), also demonstrate antifungal activity through inhibition of *Candida* growth, with EDTA treatment inducing distinct structural alterations in the *C. albicans* cell wall (Pugh and Cawson, 1980; Sen et al., 2000; Ates et al., 2005). The antifungal properties of EDTA have been attributed to its calcium chelation properties, with calcium ions shown to have a critical role in morphogenesis, adhesion and pathogenesis in *Candida* spp. (Holmes et al., 1991; Klotz et al., 1993; Gil et al., 1994). EDTA treatment has also been shown to inhibit biofilm formation (Ramage et al., 2007), reduce fungal metabolic activity in mature (72 h) biofilms (Casalinuovo et al., 2017) and kill 90-99.5% of *C. albicans* and *C. tropicalis* biofilm cell populations at concentrations of ≥ 2 mM (Harrison et al., 2007). Furthermore, when EDTA was combined with fluconazole, the structure of the resultant biofilm was greatly modified (yielding a more sparse biofilm with wider water channels) compared to biofilms treated with fluconazole alone (Casalinuovo et al., 2017). The similar changes in the biofilm architecture seen in our study suggests that the enhanced action of nystatin by OligoG could also be due to chelation of calcium. Tøndervik et al. (2014) revealed significant reductions in vegetative cell growth and an inhibitory effect on hyphal growth of *C. albicans* with OligoG treatment; findings similar to studies showing candidal growth reductions (Pugh and Cawson, 1980) and blocked hyphal development in *C. albicans* (Gil et al., 1994) when treated with EDTA.

The results shown in this and previous studies (Tøndervik et al., 2014; Pritchard et al., 2017), together with the excellent safety profile and high tolerability of OligoG proven through clinical trials, (NCT02157922; NCT02453789; Van Koningsbruggen-Rietschel et al., 2020; Fischer et al., 2022), clearly demonstrate the clinical utility of the combination treatment (OligoG/nystatin) to treat biofilm-related candidal infections, especially those on in-dwelling silicone devices such as voice box prostheses and catheters, as an aid to increase device lifespan and reduce the risk of nosocomial infections (Nobile and Johnson, 2015; Andes et al., 2012; Eix and Nett, 2020). This study also highlights the possible role of OligoG in more appropriate management of other candidal-related infections currently being treated with nystatin alone, such as topical treatments for mastitis (Douglas, 2021), skin infections, and oro-pharyngeal candidiasis (Kojic and Darouiche, 2004; Pappas et al., 2004; Pappas et al., 2016) and also the prophylaxis and treatment of surgical/trauma ICU patients (Giglio et al., 2012). In conclusion, non-toxic therapies such as OligoG, have significant potential for use alongside conventional antifungal treatments in the topical management of biofilm-related *Candida* infections.

## Data availability statement

The original contributions presented in the study are included in the article/**Supplementary Material**. Further inquiries can be directed to the corresponding author.

## Author contributions

Funding acquisition: DT, KH, MP; Conceived and designed experiments: LP, DT, KH, MP, SQ, DO, CP, AO; Performed the experiments: JA, LP, SG, SQ, CR; Analysed the data: JA, LP, SG, SQ; Contributed reagents/materials/analysis tools: PR, LF; Wrote and edited the paper: LP, JA, SQ, KH, MP, CR, DT; All authors read and approved the final manuscript.
