# Supplementary material for: Alginate oligosaccharides enhance the antifungal activity of nystatin against candidal biofilms
Source: Front Cell Infect Microbiol. 2023 Jan 31;13:1122340. doi: 10.3389/fcimb.2023.1122340 (PMC9927220; doi:10.3389/fcimb.2023.1122340)
Supplement: Supplementary file 1 [file DataSheet_1.docx]

**Supplementary Materials and Methods**

**Silicone sheet preparation and characterization**

Contact profilometry and atomic force microscopy (AFM) imaging were performed to assess the surface roughness of the silicone material following autoclaving. The silicone pieces were scanned using a Surftest SV-2000 (Mitutoyo UK Ltd) consisting of a 20 µm diamond stylus, where the average surface roughness (Ra) was determined. Silicone samples were also analyzed using a BioScope Catalyst AFM (Bruker Instruments, USA). Sample topography was studied in Tapping Mode using RTESP cantilevers (Bruker Instruments, USA), with a nominal spring constant of 40 N/m and a nominal resonance frequency of 300 kHz. The roughness Ra was measured through 25 μm^2^ sub-areas of a 400 μm^2^ AFM image area, with more than 50 roughness values for each sample type recorded. The roughness subroutine in the Nanoscope Analysis software v1.50 was used.

# Supplementary Results

**Silicone Sheet Roughness Measurements**

The surface of the clinical grade silicone substrate was characterized using contact profilometry and AFM imaging to generate meso- and nanoscale surface roughness measurements, especially as surface roughness may influence microbial attachment and subsequent biofilm formation (Zheng et al., 2021). Contact profilometry measurements showed that the bulk scale surface roughness of the silicone was 0.95 ± 0.07 µm. In addition, AFM measurements demonstrated the nanoscale surface roughness of the silicone surface was 17 ± 0.07 nm.

**Reference**

Zheng, S., Bawazir, M., Dhall, A., Kim, H-E., He, L., Heo, J., Hwang, G. (2021). Implication of Surface Properties, Bacterial Motility, and Hydrodynamic Conditions on Bacterial Surface Sensing and Their Initial Adhesion. *Front. Bioeng. Biotechnol.* 9, 643722. doi:10.3389/fbioe.2021.643722
