# Supplementary material for: Alginate oligosaccharides enhance the antifungal activity of nystatin against candidal biofilms
Source: Front Cell Infect Microbiol. 2023 Jan 31;13:1122340. doi: 10.3389/fcimb.2023.1122340 (PMC9927220; doi:10.3389/fcimb.2023.1122340)
Supplement: Supplementary file 2 [file Table_1.docx]

**SUPPLEMENTARY TABLE 1.** P values of Tukey’s post-hoc comparison testing of ATP cellular viability assay for the data shown in Figure 5.

| **Isolate** | **Control v OligoG** | **Control v NYS** | **Control v OligoG/NYS** | **OligoG v NYS** | **OligoG v OligoG/NYS** | **NYS v OligoG/NYS** |
| --- | --- | --- | --- | --- | --- | --- |
| *C. albicans* ATCC 90028 | <0.0001 | 0.0442 | <0.0001 | <0.0001 | 0.1567 | <0.0001 |
| *C. albicans* GBJ 13/4A | <0.0001 | <0.0001 | <0.0001 | <0.0001 | 0.0006 | <0.0001 |
| *C. albicans* SC5314 | <0.0001 | <0.0001 | <0.0001 | <0.0001 | 0.2334 | <0.0001 |
| *C. albicans* CCUG 39343 | <0.0001 | <0.0001 | <0.0001 | <0.0001 | <0.0001 | <0.0001 |
| *C. albicans* 480/00 | <0.0001 | <0.0001 | <0.0001 | <0.0001 | 0.0286 | <0.0001 |
| *C. albicans* PB1/93 | <0.0001 | <0.0001 | <0.0001 | <0.0001 | 0.0043 | <0.0001 |
| *C. albicans* Lr1/93 | <0.0001 | 0.0366 | <0.0001 | <0.0001 | 0.6671 | <0.0001 |
| *C. albicans* Ptr/94 | 0.0001 | 0.5431 | <0.0001 | 0.006 | 0.8348 | 0.0006 |
| *C. auris* NCPF 8971 | <0.0001 | 0.4618 | <0.0001 | <0.0001 | 0.072 | <0.0001 |
| *C. dubliniensis* 40/01 | 0.0394 | 0.0431 | 0.1916 | <0.0001 | 0.8697 | 0.0002 |
| *C. glabrata* ATCC 2001 | <0.0001 | 0.0421 | <0.0001 | 0.0001 | <0.0001 | <0.0001 |
| *C. parapsilosis* W23 | 0.0002 | 0.3739 | 0.0056 | <0.0001 | 0.5907 | <0.0001 |
| *C. tropicalis* 519468 | <0.0001 | 0.0054 | <0.0001 | <0.0001 | 0.5956 | <0.0001 |

NYS, nystatin. Significant reductions in ATP values are highlighted in grey (p < 0.05)
