# Supplementary figures and images for: Alginate oligosaccharides enhance the antifungal activity of nystatin against candidal biofilms
Source: Front Cell Infect Microbiol. 2023 Jan 31;13:1122340. doi: 10.3389/fcimb.2023.1122340 (PMC9927220; doi:10.3389/fcimb.2023.1122340)

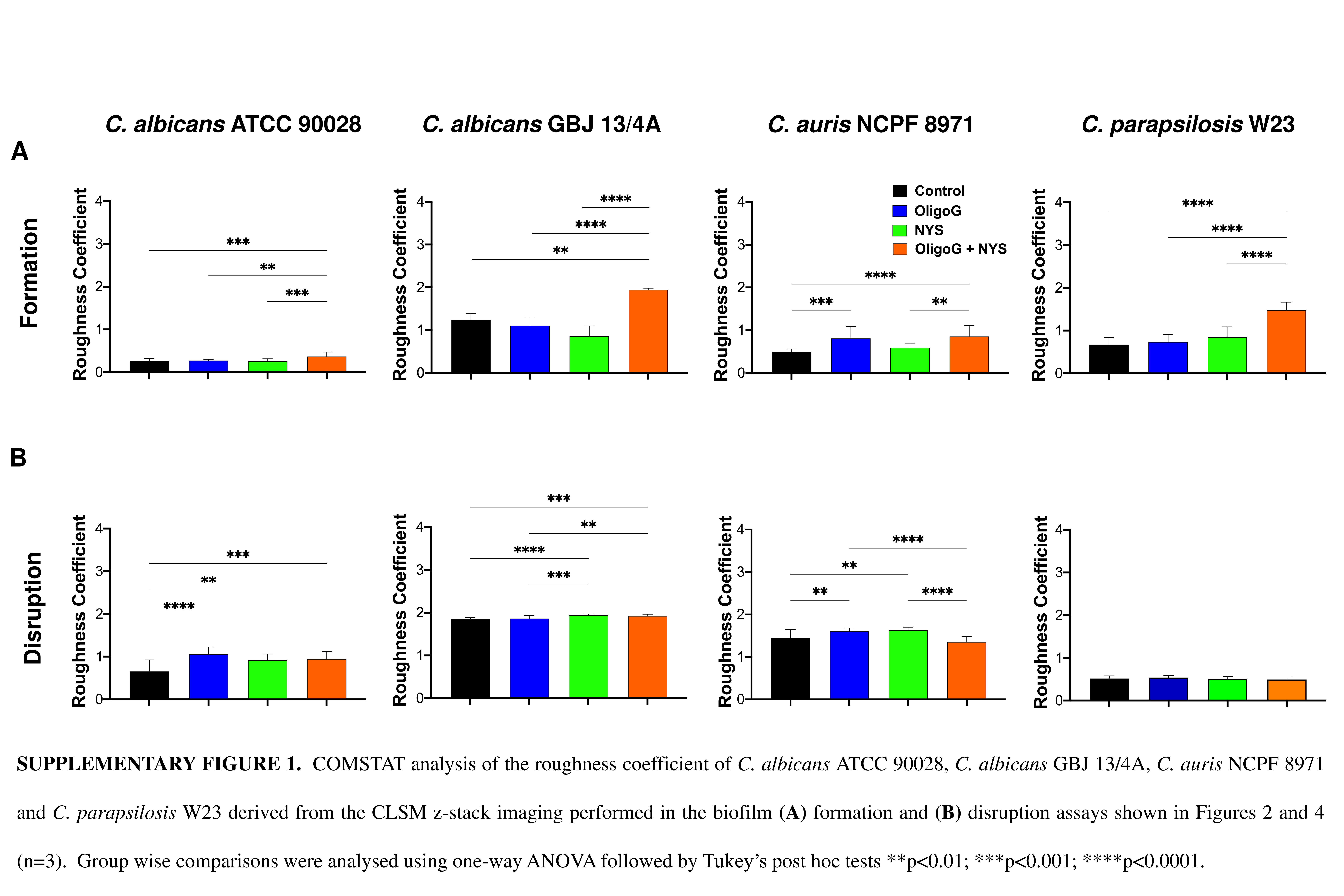

Supplement: Supplementary file 3 [file Image_1.tif]
